# Targeting Bruton tyrosine kinase using non-covalent inhibitors in B cell malignancies

**DOI:** 10.1186/s13045-021-01049-7

**Published:** 2021-03-06

**Authors:** Danling Gu, Hanning Tang, Jiazhu Wu, Jianyong Li, Yi Miao

**Affiliations:** 1 Department of Hematology, The First Affiliated Hospital of Nanjing Medical University, Jiangsu Province Hospital, Nanjing, 210029 China; 2 Key Laboratory of Hematology of Nanjing Medical University, Nanjing, 210029 China; 3 Pukou CLL Center, Nanjing, 210000 China

**Keywords:** BTK, B cell malignancies, Ibrutinib, C481 mutations, Non-covalent inhibitors

## Abstract

B cell receptor (BCR) signaling is involved in the pathogenesis of B cell malignancies. Activation of BCR signaling promotes the survival and proliferation of malignant B cells. Bruton tyrosine kinase (BTK) is a key component of BCR signaling, establishing BTK as an important therapeutic target. Several covalent BTK inhibitors have shown remarkable efficacy in the treatment of B cell malignancies, especially chronic lymphocytic leukemia. However, acquired resistance to covalent BTK inhibitors is not rare in B cell malignancies. A major mechanism for the acquired resistance is the emergence of BTK cysteine 481 (C481)  mutations, which disrupt the binding of covalent BTK inhibitors. Additionally, adverse events due to the off-target inhibition of kinases other than BTK by covalent inhibitors are common. Alternative therapeutic options are needed if acquired resistance or intolerable adverse events occur. Non-covalent BTK inhibitors do not bind to C481, therefore providing a potentially effective option to patients with B cell malignancies, including those who have developed resistance to covalent BTK inhibitors. Preliminary clinical studies have suggested that non-covalent BTK inhibitors are effective and well-tolerated. In this review, we discussed the rationale for the use of non-covalent BTK inhibitors and the preclinical and clinical studies of non-covalent BTK inhibitors in B cell malignancies.

## Background

**Fig. 1:**
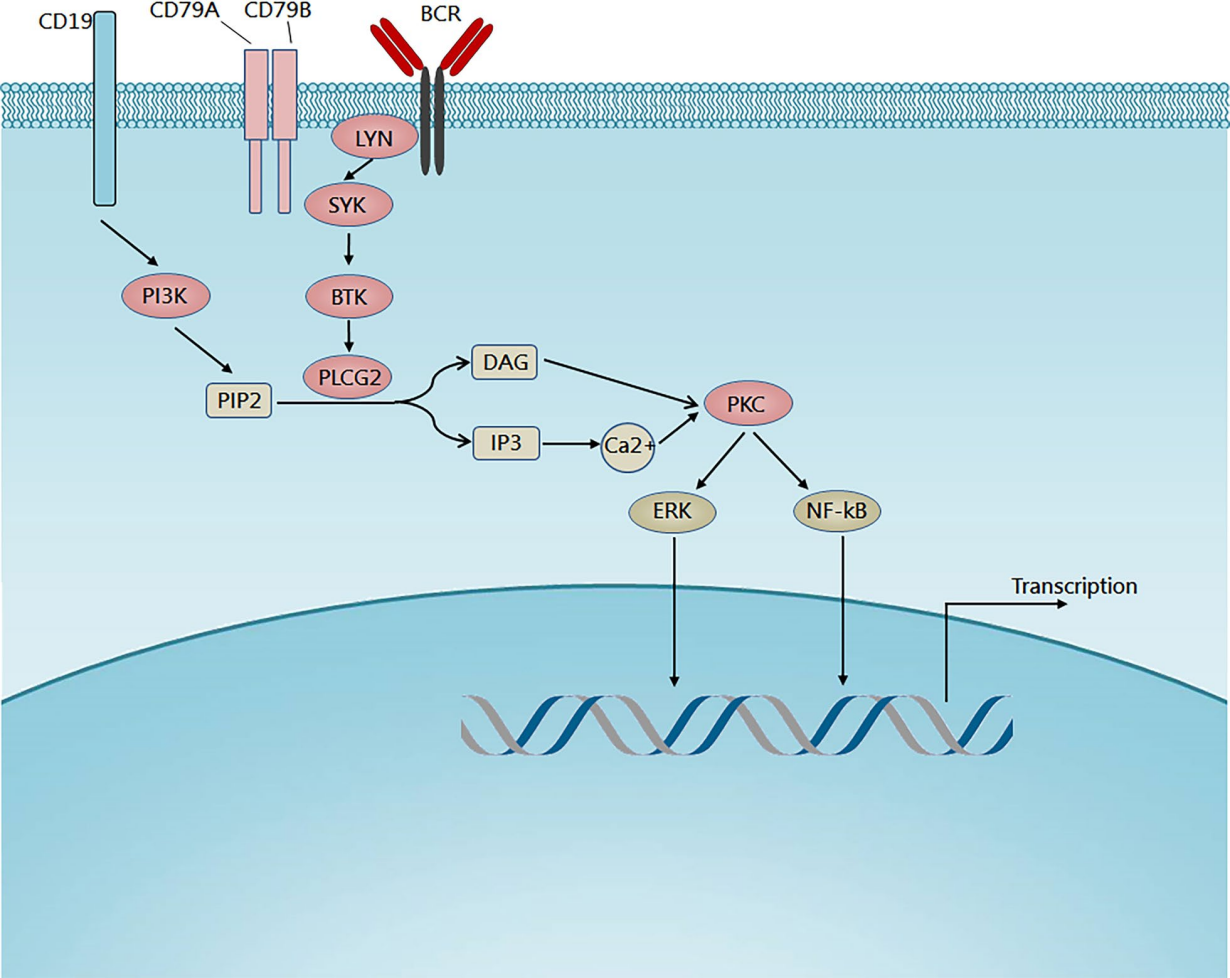
BCR signaling. The binding of antigens to the B cell receptor leads to the phosphorylation of the intracellular immunoreceptor tyrosine-based activation motifs (ITAMs) of CD79A and CD79B. The phosphorylation of CD79A/CD79B initiates SYK activation, which then results in BTK activation and subsequent PLCG2 activation. This signal cascade ultimately leads to the activation of NF-κB and MAPK/ERK pathways, contributing to the survival and proliferation of CLL cells. BTK and PLCG2 mutations are detected in BTK inhibitor-resistant CLL cases

B cell receptor (BCR) signaling plays a crucial role in B cell development and adaptive immune response and also contributes to the pathogenesis of different types of B cell malignancies [1, 2]. The BCR signaling cascades involve several essential kinases, including spleen tyrosine kinase (SYK), Bruton tyrosine kinase (BTK), and phosphatidylinositol-3-kinase (PI3K) (Fig. 1) [1]. Briefly, BCR ligation by antigen leads to phosphorylation of immunoreceptor tyrosine-based activation motif (ITAM) of CD79A and CD79B, thereby recruiting SYK [1]. SYK then phosphorylates and activates BTK. BTK activation initiates further downstream signaling pathways including nuclear factor-κB (NF-κB) pathway, MAPK/ERK pathway, and other pathways.

**Fig. 2:**
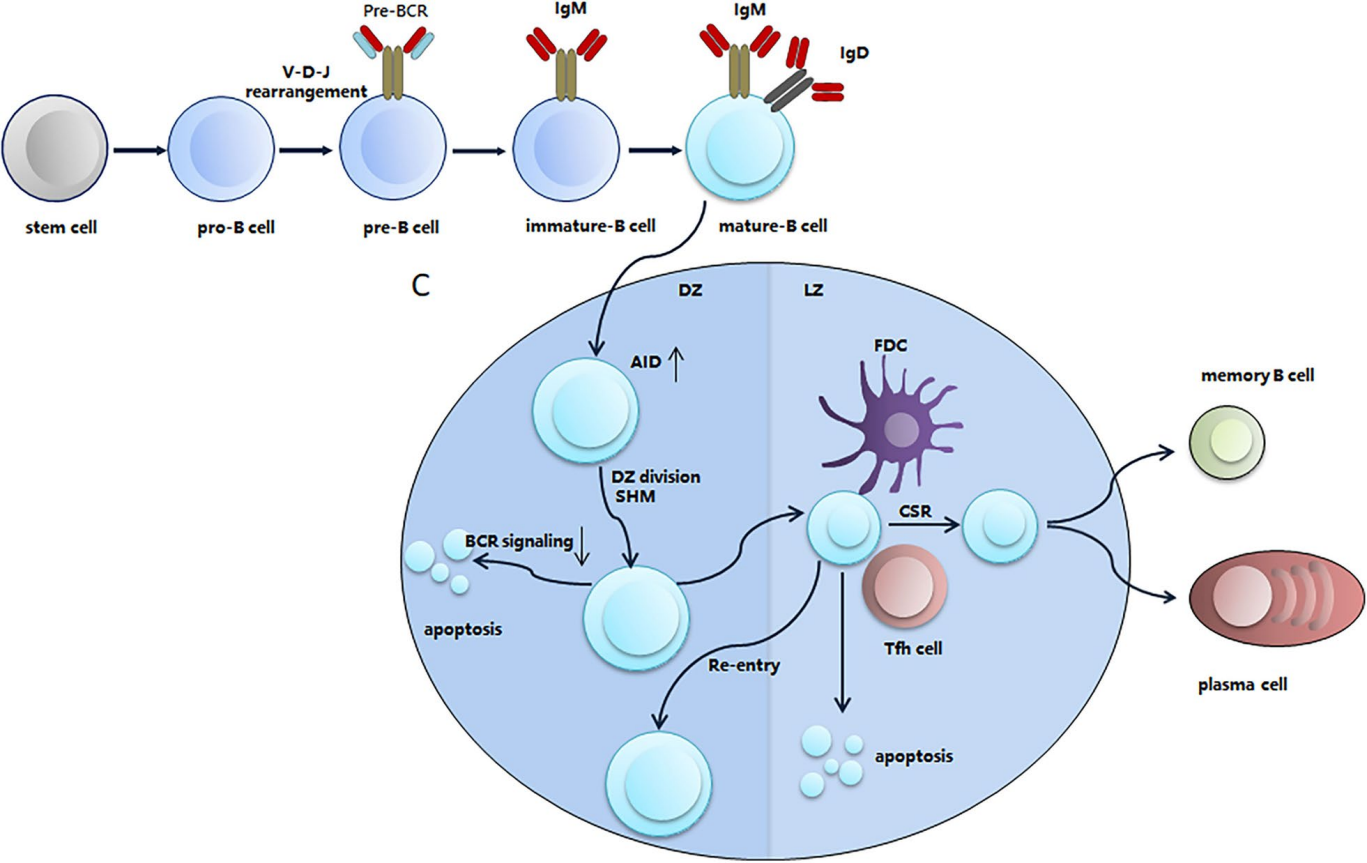
BCR in B cell development. In the bone marrow, progenitor B (pro-B) cells undergo the rearrangement and development of immunoglobulin heavy-chain variable (V), diversity (D), and joining (J) gene segments to form the pre-BCR. The pre-BCR is an immature form of the BCR providing signals for survival, proliferation, and cellular differentiation. After light-chain gene rearrangement occurs, immature B cells express BCR, leave the bone marrow, and mature in the periphery. Mature B cells undergo somatic hypermutation (SHM) driven by the expression of activation-induced cytidine deaminase (AID) in the germinal center (GC) to complete BCR affinity maturation and antibody diversification. The genotoxic stress induced by SHM may lead to the apoptosis of these B cells. However, the continuous BCR signaling could provide pro-survival signals for these B cells, thereby preventing them from apoptosis. Therefore, B cells deficient in Bruton tyrosine kinase tend to undergo apoptosis during the development. The B cells that have completed affinity maturation and antibody diversification then undergo class-switch recombination (CSR), after which they develop into memory B cells with high-affinity BCRs or plasma cells secreting antibodies. DZ, dark zone; LZ, light zone; FDC, follicular dendritic cell; Tfh cell, T follicular helper cell

**Fig. 3:**
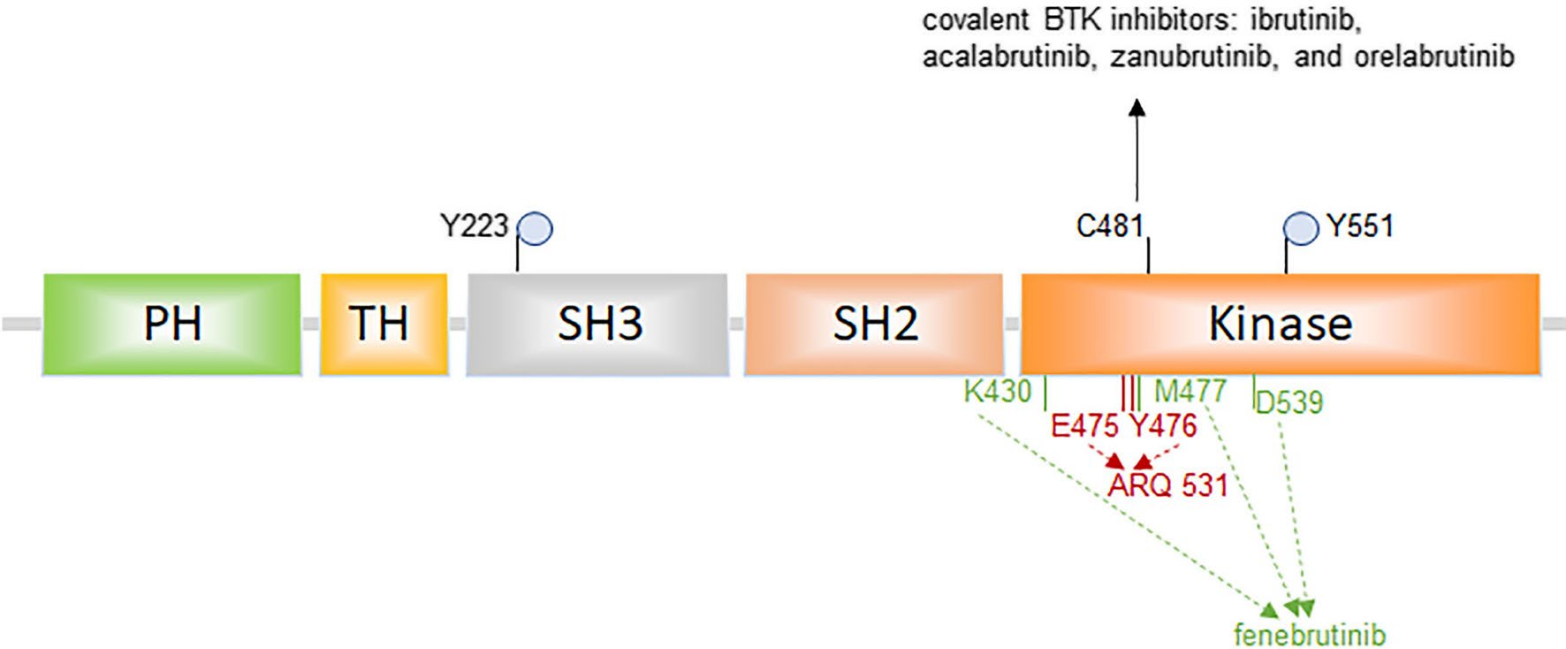
The structural diagram of Bruton tyrosine kinase (BTK). The BTK protein is a 77 kDa protein of 659 amino acids, which contains five different protein interaction domains. There are two critical tyrosine phosphorylation sites, Y223 in the SH3 domain and Y551 in the kinase domain. BTK inhibitors bind to the BTK kinase domain and blocks the catalytic activity of BTK. Currently available covalent BTK inhibitors, including ibrutinib, acalabrutinib, zanubrutinib, and orelabrutinib, selectively bind to C481 residue in the allosteric inhibitory segment of the BTK kinase domain. The non-covalent BTK inhibitors do not bind to C481. For example, ARQ 531 binds to BTK by forming hydrogen bonds with E475 and Y476 residues [56]. Fenebrutinib forms hydrogen bonds with K430, M477, and D539 residues [50]

The essential role of BTK in BCR signaling makes it an ideal target for suppressing BCR signaling. BTK was originally identified as a non-receptor protein tyrosine kinase in 1993 [3, 4]. BTK is a member of the Tec family kinases, which contain interleukin-2-inducible T cell kinase (ITK), tyrosine kinase expressed in hepatocellular carcinoma (TEC), resting lymphocyte kinase (RLK), and bone marrow expressed kinase (BMX)[5]. *BTK* loss-of-function mutations result in X-linked agammaglobulinemia, a type of immunodeficiency that is characterized by the lack of mature B cells and immunoglobulins and consequent opportunistic infections in young boys [3, 4], highlighting the importance of BTK in B cell development and humoral immunity. The process of B cell development and the role of BCR in this process are described in Fig. 2. BTK comprises five different protein interaction domains, which include an amino-terminal pleckstrin homology (PH) domain, a proline-rich TEC homology (TH) domain, SRC homology (SH) domains SH2 and SH3, and a kinase domain (Fig. 3) [6, 7]. BTK has two critical tyrosine phosphorylation sites, Y223 in the SH3 domain and Y551 in the kinase domain. During BCR signaling, the phosphorylation by SYK at Y551 enhances the catalytic activity of BTK and initiates subsequent Y223 autophosphorylation [7]. BTK inhibitors, including covalent and non-covalent inhibitors, bind to the BTK kinase domain and block the catalytic activity of BTK, thereby suppressing subsequent Y223 autophosphorylation [6]. Several covalent BTK inhibitors, including ibrutinib, acalabrutinib, zanubrutinib, and orelabrutinib, have been developed for targeting BTK in B cell malignancies [8–11]. These covalent inhibitors have shown remarkable efficacy in B cell malignancies, including chronic lymphocytic leukemia (CLL), mantle cell lymphoma (MCL), Waldenström's macroglobulinemia (WM), and marginal zone lymphoma (MZL). However, treatment failure due to drug resistance or adverse events (AEs) is not rare in clinical practice. Overcoming drug resistance and reducing severe AEs are of vital importance to improve the outcomes of patients. Using non-covalent BTK inhibitors could be a strategy for overcoming drug resistance and reducing adverse events. In this review, we discussed the rationale of the application of non-covalent BTK inhibitors and their preclinical and clinical studies in B cell malignancies.

## Covalent BTK inhibitors

**Fig. 4:**
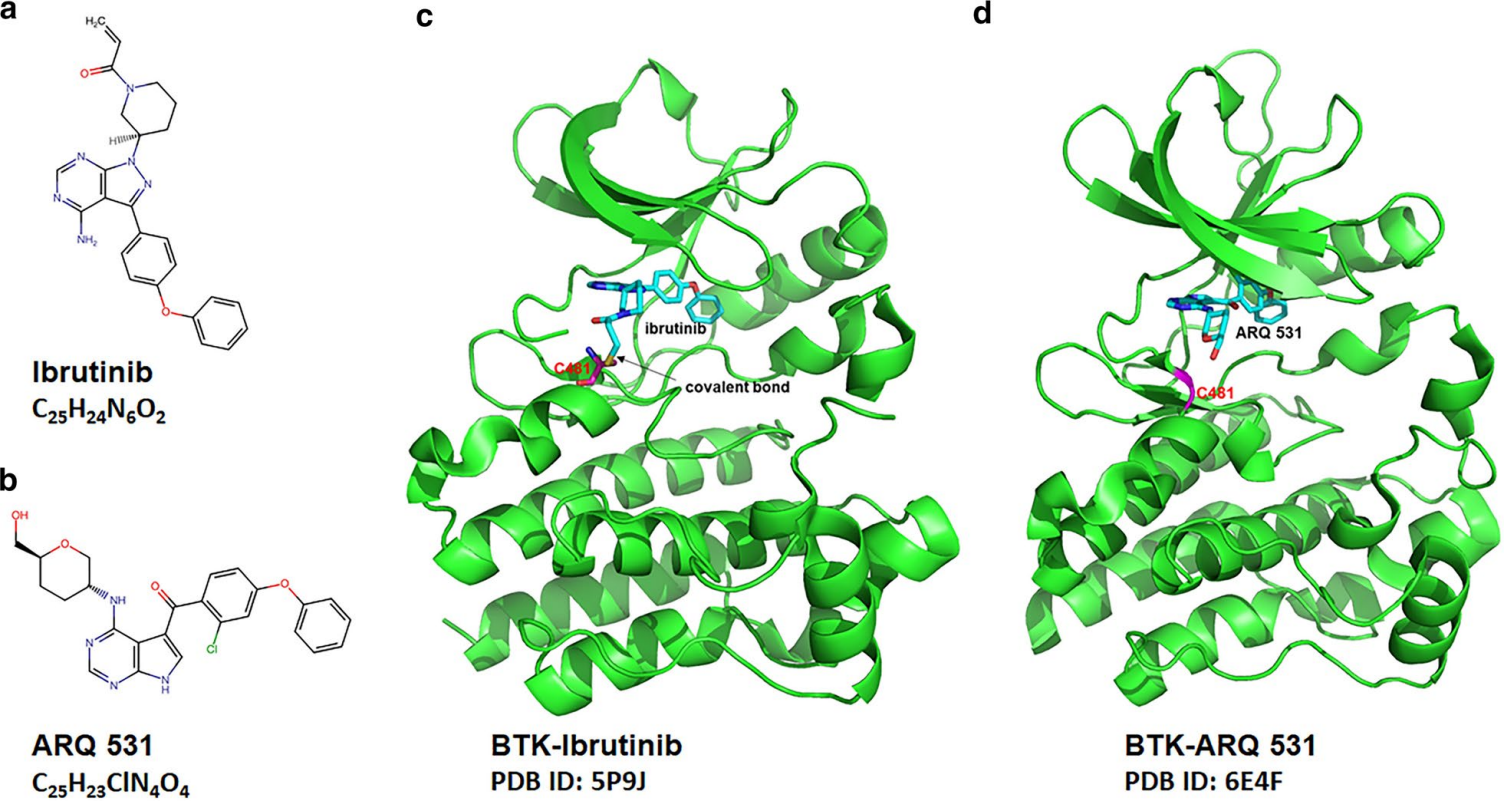
Mechanisms for the action of representative BTK inhibitors. **a** Chemical structure of the covalent BTK inhibitor ibrutinib. **b** Chemical structure of the non-covalent BTK inhibitor ARQ 531. **c** Ibrutinib covalently binds to BTK cysteine 481 (C481), competes with ATP in the ATP binding pocket, and inhibits autophosphorylation of BTK. The action of ibrutinib requires its binding to BTK C481, and BTK C481 mutations abrogate the binding of ibrutinib and lead to the resistance to ibrutinib. **d** ARQ 531 non-covalently interacts with BTK, occupies the ATP binding pocket, and inhibits BTK autophosphorylation. The effect of ARQ 531 does not require its binding to BTK C481. Therefore, ARQ 531 remains active in patients with BTK C481 mutations. **c**, **d** were made by using PyMOL 0.99

Several covalent BTK inhibitors, which include ibrutinib, acalabrutinib, and zanubrutinib, have been tested in clinical trials and have been approved for treating patients with B cell malignancies. The results of phase III studies with covalent BTK inhibitors are summarized in Table 1. Ibrutinib (PCI-32765) is an irreversible, highly potent small molecule BTK inhibitor that covalently binds to cysteine481 (C481) in the active site of BTK and blocks the full activation of BTK by inhibiting its autophosphorylation at tyrosine 223, suppressing signaling downstream of BTK (Fig. 4a, c) [12]. Ibrutinib has shown remarkable efficacy in CLL, MCL, WM, and MZL [8, 10, 13–16]. The use of ibrutinib has revolutionized the treatment of B cell malignancies, especially CLL. Several phase III trials have demonstrated the superiority of ibrutinib monotherapy in both relapsed/refractory and treatment-naïve CLL patients [8, 14–18]. A recent pooled analysis of four clinical trials showed that first-line ibrutinib treatment resulted in high long-term efficacy (progression-free survival 79% and overall survival 88% at 48 months) in CLL patients with TP53 aberrations, a group of patients with historically poor prognosis [19]. Combinations of ibrutinib with other novel drugs or regimens result in more profound responses and much higher rates of minimal residual disease (MRD) negativity [20, 21]. Other covalent BTK inhibitors, including acalabrutinib and zanubrutinib [9, 22], have also shown promising efficacy in B cell malignancies. Although the covalent BTK inhibitors are effective in B cell malignancies, resistance to these BTK inhibitors, including primary and acquired resistance, is frequent in patients with B cell malignancies [23]. The major mechanisms for the resistance to the covalent BTK inhibitors are summarized in Table 2. Here, we briefly discussed the mechanisms for the acquired resistance to covalent BTK inhibitors.

**Table 1:** Published phase III studies of covalent BTK inhibitors in B cell malignancies

Drug	Number	Patients	Regimen	Response rates	Survival
Ibrutinib	NCT02165397	TN or R/R WM (*n* = 150)	IR versus placebo + RTX	IR: MR 72%, CR 3%, VGPR 23%; Placebo plus RTX: MR 32%, CR 1%, VGPR 4%	IR: PFS 68% and OS 86% at 54 mon; placebo plus RTX: PFS 25% and OS 84% at 54 mon [72, 73]
Ibrutinib	NCT01611090	R/R CLL/SLL (*n* = 578)	Ibrutinib + BR versus placebo + BR	Ibrutinib + BR: ORR 87.2%, CR/CRi 38.1%; placebo + BR: ORR 66.4%, CR/CRi: 8.0%	Ibrutinib + BR: PFS 68.0% and OS 81.6% at 3 yr; Placebo + BR: PFS 13.9% and OS 72.9% at 3 yr [74]
Ibrutinib	NCT01578707	R/R CLL/SLL (*n* = 391)	Ibrutinib versus ofatumumab	Ibrutinib: ORR 91%, CR/CRi 11%; ofatumumab: ORR 4.1%, CR/CRi 1%;	Ibrutinib: median PFS 44.1 mon and median OS 67.7 mon; ofatumumab: median PFS 8.0 mon and median OS 65.1 mon [17, 18]
Ibrutinib	NCT01722487 NCT01724346	TN CLL/SLL, age ≥ 65 yr (*n* = 269)	Ibrutinib versus chlorambucil	Ibrutinib: ORR 92%, CR/CRi 30%; chlorambucil: ORR: 37%, CR/CRi 2%	Ibrutinib: PFS 70% and OS 83% at 5 yr; chlorambucil: PFS 12% and OS 68% at 5 yr [8]
Ibrutinib	NCT01886872	TN CLL, age ≥ 65 yr (*n* = 547)	Ibrutinib versus IR versus BR	Ibrutinib: ORR 93%, CR 7% IR: ORR: 94%; CR:12% BR: ORR: 81%; CR:26%	Ibrutinib: PFS 87% and 90% at 2 yr; IR: PFS 88% and OS 94% at 2 yr; BR: PFS 74% and OS 95% at 2 yr [16]
Ibrutinib	NCT02048813	TN CLL/SLL, age ≤ 70 yr (*n* = 529)	IR versus FCR	IR: ORR 95.8%, CR/CRi 17.2% FCR: ORR 81.1%, CR/CRi 30.3%	IR: PFS 89.4% and OS 98.8% at 3 yr FCR: PFS 72.9% and OS: 91.5% at 3 yr [15]
Ibrutinib	NCT02264574	TN CLL/SLL (*n* = 229)	Ibrutinib + GA-101 versus chlorambucil + GA-101	Ibrutinib + GA-101: ORR 88%, CR/CRi 19%; chlorambucil + GA-101: ORR 73%, CR/CRi 8%	Ibrutinib + GA-101: PFS 79% and OS 86% at 30 mon; chlorambucil + GA-101: PFS: 31% and OS 85% at 30 mon [14]
Ibrutinib	NCT01646021	R/R MCL (*n* = 280)	Ibrutinib versus temsirolimus	Ibrutinib: ORR 72%, CR 19%; temsirolimus: ORR 40% CR 1%	Ibrutinib: median PFS 15.6 mon and median OS 30.3 mon; temsirolimus: median PFS 6.2 mon and median OS 23.5 mon [13]
Acalabrutinib	NCT02475681	TN CLL (*n* = 535)	Acalabrutinib versus acalabrutinib + GA-101 versus chlorambucil + GA-101	Acalabrutinib: ORR 94%, CR/CRi 24%; acalabrutinib + GA-101: ORR 85%, CR/CRi 1%; chlorambucil + GA-101: ORR 79%, CR/CRi 5%;	Acalabrutinib: PFS 87% at OS 95% at 24 mon; acalabrutinib + GA-101: PFS 93% and OS 95% at 24 mon; chlorambucil + GA-101: PFS 47% and OS 92% at 24 mon [9]
Ibrutinib zanubrutinib	NCT03053440	TN or R/R WM with MYD88^L265P^ (*n* = 201)	Ibrutinib versus zanubrutinib	Ibrutinib: MR 78%, VGPR: 19% zanubrutinib: MR 77%, VGPR 28%	Ibrutinib: PFS 84% and OS 93% at 18 mon; zanubrutinib: PFS 85% and OS 97% at 18 mon [10]
Ibrutinib	NCT01855750	TN non-GCB DLBCL (*n* = 838)	Ibrutinib + R-CHOP versus R-CHOP	Ibrutinib + R-CHOP: ORR 87.3%, CR 67.3%; R-CHOP: ORR 93.1%, CR 68.0%	For age < 60 yr: Ibrutinib + R-CHOP: PFS 77.4% and OS 93.2% at 3 yr; R-CHOP: PFS 66.3% and OS 80.9% at 3 yr For age ≥ 60 yr: Ibrutinib + R-CHOP: PFS 66.8% and OS 76.6% at 3 yr; R-CHOP: PFS 69.6% and OS 81.7% at 3 yr [75]

*TN* treatment naïve, *R/R* relapsed/refractory, *WM* Waldenstrom's macroglobulinemia, *IR* ibrutinib and rituximab, *RTX* rituximab, *MR* major response, *CR* complete response, *CRi* complete response with incomplete hematologic recovery, *VGPR* very good partial response, *PFS* progression free survival, *OS* overall survival, *mon* months, *CLL/SLL* chronic lymphocytic leukemia/small lymphocytic lymphoma, *BR* bendamustine and rituximab, *ORR* overall response rate, *yr* years, *FCR* fludarabine, cyclophosphamide, and rituximab, *MCL* mantle cell lymphoma, *non-GCB* Non-germinal center B cell, *R-CHOP* rituximab plus cyclophosphamide, doxorubicin, vincristine, and prednisone

**Table 2:** Resistance mechanisms to BTK inhibitors in B cell malignancies

B cell malignancy subtype	Covalent BTK inhibitors	Mechanisms underlying resistance
*Primary resistance*		
MCL	Ibrutinib	Mutations involving NF-κB pathway: A20 mutations, TRAF2 mutations, BIRC3 mutations or BIRC2 mutations, RELA E39Q mutation, and others [76, 77] Sustained PI3K/AKT/mTOR activation [28, 78] Tumor microenvironment [79] Metabolic reprogramming toward oxidative phosphorylation and glutaminolysis [80] CCND1 mutation [81]
WM	Ibrutinib	CXCR4 WHIM-like mutations [82]
DLBCL	Ibrutinib	PIM1 mutation [83] PI3K/AKT activation [84] MAPK activation [84] Aberrations activating NF-κB pathway: CARD11 mutation, A20 aberrations [85] High expression of PDGFD [86]
*Acquired resistance*		
CLL/SLL	Ibrutinib	BTK C481 and T474 mutations [24, 87, 88] PLCG2 mutations (R665W, S707, L845F, and others) [87] del(8p) [89]
CLL/SLL	Acalabrutinib	BTK C481 mutations and T474I mutation, PLCG2 mutations [29]
CLL/SLL	Zanubrutinib	BTK Leu528Trp mutation and C481 mutation [31]
MCL	Ibrutinib	BTK C481S mutation [28] PLCG2 mutations [76] CARD11 mutation [76] Tumor microenvironment [79]
WM	Ibrutinib	BTK C481 mutations [26] PLCG2 Tyr495His mutation [26]
MZL	Ibrutinib	BTK C481S mutation [90] PLCG2 R665W [90]
DLBCL	Ibrutinib	BTK C481S mutation [91]

*BTK* Bruton tyrosine kinase, *MCL* mantle cell lymphoma, *WM* Waldenstrom's macroglobulinemia, *DLBCL* diffuse large B cell lymphoma, *CLL/SLL* chronic lymphocytic leukemia/small lymphocytic lymphoma, *MZL* marginal zone lymphoma

### Acquired resistance to BTK inhibitors

Despite the clinical success ibrutinib has achieved, it should be noted that CLL progression or Richter transformation (RT) occurs in a subset of CLL patients administrated with ibrutinib [24]. Most of the patients who experience CLL relapse have BTK C481 mutations and less commonly PLCG2 mutations [24]. The BTK C481 mutations, most of which are BTK C481S, involve the cysteine where ibrutinib binding occurs, rendering ibrutinib unable to inhibit BTK and downstream pathways. BTK C481 mutations are also detected in approximately 30% of patients who underwent RT on ibrutinib, suggesting BTK C481 mutations could be involved in mediating RT on ibrutinib [25]. Ibrutinib resistance has also been observed in WM patients with active ibrutinib therapy. BTK C481 mutations are commonly observed in WM patients who experienced progression, suggesting BTK C481 mutations are also responsible for ibrutinib resistance in WM [26]. Both primary resistance (10.2–35%) and acquired resistance (17.5–54%) to ibrutinib are common in ibrutinib-treated patients with MCL [27]. Acquired BTK C481S mutation is detected in a small proportion of MCL patients who relapsed after ibrutinib therapy [28].

Disease progression due to drug resistance has also been reported in patients treated with other covalent inhibitors. According to the study by Woyach et al., with a median follow-up of 47.5 months, CLL relapse occurred in 17 of 105 CLL patients on acalabrutinib therapy [29]. BTK C481 mutations were identified in 11 of these 16 patients, indicating that the mechanism for acalabrutinib resistance is similar to that for ibrutinib resistance [29]. Thirty-one percent of R/R MCL patients on acalabrutinib had disease progression with a median follow-up of only 15.2 months. The mechanisms responsible for acalabrutinib resistance in MCL have not been characterized yet [30]. The acquired resistance also occurs in CLL patients treated with zanubrutinib, although at a relatively low rate [22]. CLL patients progressing on zanubrutinib had concurrent BTK Leu528Trp and BTK C481 mutations [31]. The co-occurrence of BTK Leu528Trp and BTK C481 mutations suggests these mutations may cooperate in mediating resistance to zanubrutinib.

### Adverse events of covalent BTK inhibitors

In addition to ibrutinib resistance, ibrutinib intolerance is also a concern in patients treated with ibrutinib, frequently leading to ibrutinib discontinuation [32, 33]. Some kinases also harbor a modifiable cysteine residue that is homologous to C481 in BTK; therefore, these kinases could also be inhibited by ibrutinib [34]. A variety of receptor tyrosine kinases and non-receptor tyrosine kinases, including EGFR, ITK, TEC, ERBB4, BMK, JAK3, and HER2 [35], are covalently inhibited by ibrutinib. The inhibition of these non-BTK kinases accounts for part of the AEs related to ibrutinib. The common AEs in patients treated with ibrutinib include diarrhea, bleeding, atrial fibrillation, infection, and others, and serious AEs may cause discontinuation. According to a pooled analysis, diarrhea occurred in approximately half of the CLL patients treated with ibrutinib and 5% of patients had grade 3 diarrhea [36]. Ibrutinib-related diarrhea could be attributed to the inhibition of EGFR [37]; however, the exact mechanism remains to be determined. Bleeding/bruising events occurred in 55% of CLL patients on ibrutinib therapy, and 7.6% of patients experienced major hemorrhage events [36]. The inhibition of BTK and other Tec family kinases impairs glycoprotein VI signalings, suppressing platelet aggregation, and thereby contributing to bleeding events [38–40]. Approximately 11% of CLL patients developed atrial fibrillation during ibrutinib monotherapy, and 5% of patients developed grade 3 atrial fibrillation [36]. It should be noted that the frequency of atrial fibrillation is various in different studies, with younger patients having a lower rate of atrial fibrillation (7.4% in the E1912 study) [15]. Ibrutinib inhibits BTK and TEC in cardiac tissue, resulting in the downregulation of the cardiac PI3K/AKT pathway. It has been reported that reduced PI3K/AKT activity increased the susceptibility to atrial fibrillation [41]. Therefore, ibrutinib may lead to atrial fibrillation by inhibiting the PI3K/AKT pathway in cardiac tissue [42]. Infection was prevalent (83%) in CLL patients on ibrutinib therapy, and grade 3 or 4 infection occurred in 29% of patients [36]. The significantly increased risk of infection could be caused by immune impairment, which is attributed to the inhibition of ITK in T cells and BTK in macrophages [43]. Other common AEs include arthralgia, fatigue, hypertension, and rash. Consistently, the second-generation covalent BTK inhibitors, including acalabrutinib, zanubrutinib, and orelabrutinib, bind to C481 residue. Current studies have shown that the second-generation covalent BTK inhibitors have higher specificity and fewer off-target toxicities, while still cause adverse events. For instance, headache (43%) and diarrhea (39%) were very common in acalabrutinib-treated relapsed CLL patients [44]. Further, the follow-up of patients treated with these second-generation covalent BTK inhibitors is relatively short compared to those treated with ibrutinib, so that longer follow-up is warranted to observe potential adverse events.

## Non-covalent BTK inhibitors in B cell malignancies

Non-covalent BTK inhibitors do not bind to C481 (Figs. 3, 4d), therefore providing a potentially effective option to patients with B cell malignancies, including those who are resistant to covalent BTK inhibitors due to BTK C481 mutations. These non-covalent BTK inhibitors could administrate to patients who have not received covalent BTK inhibitors previously to reduce the risk of acquired resistance; meanwhile, they may also have fewer side effects caused by the off-target inhibition of kinases other than BTK. Several non-covalent BTK inhibitors have been studied and have shown promising efficacy and manageable safety profiles. The clinical trials involving non-covalent BTK inhibitors are summarized in Table 3.

**Table 3:** Clinical trials with non-covalent BTK inhibitors

Non-covalent BTK inhibitor	Phase	Patient population	Strategy	ClinicalTrials.gov ID	Status
Vecabrutinib	I/II dose escalation and expansion trial	CLL/SLL or NHL (including LPL/WM, MCL, MZL, ABC-DLBCL, FL) patients who have failed prior standard of care therapies including a BTK inhibitor where one is approved for the indication	Monotherapy	NCT03037645	Terminated
ARQ 531	I/II dose escalation and expansion trial	Dose escalation cohorts: R/R CLL/SLL, FL, MCL, MZL, and WM patients who have received ≥ 2 prior systemic therapies; expansion cohorts include R/R CLL/SLL after ≥ 2 prior systemic therapies including a covalent BTKi, with or without a C481 mutation	Monotherapy	NCT03162536	Recruiting
Fenebrutinib	Ia dose escalation trial	R/R NHL (including FL, DLBCL, MCL, PLL/WM) or CLL	Monotherapy	NCT01991184	Active, not recruiting
LOXO-305	I/II dose escalation and expansion trial	CLL/SLL or NHL patients who have failed or are intolerant to standard of care	Monotherapy; combination therapy	NCT03740529	Recruiting
LOXO-305	III	MCL patients who have been previously treated with at least one prior line of systemic therapy and were BTK inhibitor naive	Monotherapy (versus investigator choice of BTK Inhibitor)	NCT04662255	Not yet recruiting
LOXO-305	III	CLL/SLL patients who were previously treated with a covalent BTK inhibitor	Monotherapy (versus idelalisib plus rituximab or bendamustine plus rituximab)	NCT04666038	Not yet recruiting

*CLL/SLL* chronic lymphocytic leukemia/small lymphocytic lymphoma, *NHL* non-Hodgkin's lymphoma, *LPL* lymphoplasmacytic lymphoma, *WM* Waldenstrom's macroglobulinemia, *MCL* mantle cell lymphoma, *MZL* marginal zone lymphoma, *DLBCL* diffuse large B cell lymphoma, *ABC-DLBCL* the activated B cell subtype of DLBCL, *FL* follicular lymphoma, *BTK* Bruton tyrosine kinase, *R/R* relapsed and/or refractory, *PLL* prolymphocytic leukemia

### Vecabrutinib (SNS-062)

Vecabrutinib is a selective, reversible, non-covalent BTK inhibitor with nanomolar potency. In vitro studies have demonstrated vecabrutinib shows activity against both wild-type and C481S-mutated BTK. Vecabrutinib decreases surface expression of B cell activation markers and viability of primary cells from CLL patients, resulting in a phenotypic alteration that is comparable to ibrutinib [45]. Vecabrutinib also inhibits ITK but not EGFR; therefore, it may have less EGFR-mediated toxicities including rash and diarrhea [46].

A phase I study of vecabrutinib that enrolled 32 healthy participants was completed [47]. All AEs were grade 1, including headache (*n* = 5) and nausea, constipation, bronchitis, fatigue, orthostatic hypotension, and supraventricular tachycardia (*n* = 1 each), except for 1 subject who received 300 mg vecabrutinib experiencing grade 2 headache and fatigue [47]. The occupation produced by vecabrutinib and duration of BTK inhibition is encouraging [47]. A phase Ib/II dose-escalation and cohort-expansion study is ongoing in patients with relapsed/refractory advanced B cell malignancies who progressed on covalent BTK inhibitor therapy. According to the reported data, 27 patients (CLL, *n* = 21; MCL, *n* = 2; WM, *n* = 3; MZL = 1) have been treated with doses ranging from 25 to 300 mg, twice daily [48]. The maximum tolerated dose (MTD) has not been reached. Data regarding safety are available for 24 patients; the most frequent AEs included anemia (37.5%), neutropenia (25%), night sweats (25%), and headache (25%) [48]. Grade 3 drug-related AEs included alanine aminotransferase (ALT) elevation, neutropenia and worsening anemia (all in 1 patient), and leukocytosis (2 patients). Regarding efficacy, no response was observed, and 4 CLL patients, including three with BTK C481S mutation, showed stable disease. Preliminary results revealed that vecabrutinib in dose levels from 25 to 200 mg twice daily was safe in patients with B cell malignancies. The 300 mg twice daily dose level was being evaluated when the study was presented at the 2019 American Society of Hematology (ASH) meeting [48]. Vecabrutinib shows manageable safety profiles, while its efficacy in patients with B cell malignancies remains to be explored.

### Fenebrutinib (GDC-0853)

Fenebrutinib is a highly selective, reversible, non-covalent BTK inhibitor that does not bind to the C481 residue for its action and does not inhibit EGFR or ITK [49, 50]. Fenebrutinib potently inhibits BCR signaling through BTK inhibition. In vitro studies showed that fenebrutinib decreased the activation of BTK and its downstream targets upon stimulation with αIgM and reduced viability, NF-κB gene transcription, activation, and migration in CLL cells [49]. Fenebrutinib inhibits C481S BTK mutant that mediates ibrutinib resistance and is toxic to CLL cells with BTK C481S mutation. Unlike ibrutinib, which antagonizes rituximab-mediated NK cell–mediated cytotoxicity through ITK inhibition, fenebrutinib does not inhibit ITK and preserves NK cell-mediated cytotoxicity that is dependent on anti-CD20 antibodies [51]. Thus, exploration of fenebrutinib as monotherapy and in combination with anti-CD20 antibodies is promising, especially in patients with acquired resistance to ibrutinib [49, 52].

Fenebrutinib was well-tolerated with favorable safety, selectivity, and pharmacokinetic/pharmacodynamic (PK/PD) profiles in healthy volunteers [53]. A phase I study has evaluated fenebrutinib in 24 patients with relapsed/refractory B cell malignancies (CLL, *n* = 14; follicular lymphoma [FL], *n* = 4; diffuse large B cell lymphoma [DLBCL], *n* = 3; MCL, *n* = 2; prolymphocytic leukemia plus WM, *n* = 1) [54]. The enrolled patients were treated at 100, 200, or 400 mg once daily, orally. There was no dose-limiting toxicity. This phase I trial of fenebrutinib was prematurely terminated during dose escalation and the MTD was not reached. The most common AEs included fatigue (37%), nausea (33%), diarrhea (29%), thrombocytopenia (25%), and headache (20%). Eight of 24 patients had a response to fenebrutinib. Of these 8 patients who responded, an MCL patient achieved complete response (CR) and 7 CLL patients achieved partial response (PR) or PR with lymphocytosis (PR-L), including 1 of 5 heavily pretreated patients with BTK C481S mutant CLL [54]. Two additional patients with BTK C481S mutation showed a decrease in the size of target tumors (− 23% and − 44%). The median duration of response in all responding patients and patients with CLL is 3.8 months and 2.5 months, respectively. As the MTD that may provide greater BTK inhibition was not reached, the short duration of response should be interpreted cautiously. Despite this, this study provides evidence of clinical activities of reversible non-covalent BTK inhibitors in B cell malignancies [54].

#### ARQ 531

ARQ 531 is a potent, reversible inhibitor of both wild-type (WT) and mutant BTK (WT, IC50 = 0.85 nM; C481S, IC50 = 0.39 nM) with additional activities against SRC family kinases, ERK, and AKT (Fig. 4b, d). In vitro, ARQ 531 suppresses BTK-dependent functions including BCR signaling and transcription of NF-κB genes, thereby suppressing viability, cell activation, and migration of primary CLL cells [55]. ARQ 531 potently inhibits C481S-mutated BTK and downstream signaling and is also toxic to BTK C481S-mutated CLL cells [55]. In ibrutinib-resistant CLL cells with mutant PLCG2, PLCG2 can be directly activated by LYN and SYK, thereby bypassing the activation by BTK. ARQ 531 effectively inhibits signaling downstream of mutant PLCG2 and is toxic to ibrutinib-resistant CLL cells with PLCG2 mutations. In vivo studies have demonstrated the superiority of ARQ 531 over ibrutinib in mouse models of CLL and RT [56].

A phase I dose-escalation study of ARQ 531 has been completed in patients with relapsed or refractory B cell malignancies [57]. Totally, 40 patients (CLL/SLL, *n* = 26; RT, *n* = 6; DLBCL, *n* = 3; FL, *n* = 4; MCL, *n* = 1) were enrolled [57]. The enrolled patients were treated with a median of 4 prior therapies and were all previously treated with an irreversible BTK inhibitor. Most patients with CLL (22/26, 85%) had BTK C481S mutation. Doses of 5, 10, 15, 20, 30, 45, 65, and 75 mg daily were used. ARQ 531 was well tolerated, and most of the drug-related treatment emergent AEs (TEAEs) were grade 1 or 2. Partial response was achieved in 10 patients, including patients with CLL (*n* = 7), RT (*n* = 1), DLBCL (*n* = 1), and FL (*n* = 1) [57]. Patients that responded to ARQ 531 were mainly from the higher dose cohorts [65 mg daily]. ARQ 531 at 65 mg daily showed manageable safety profiles and significant anti-tumor efficacy; therefore, 65 mg daily was determined as the recommended phase 2 dose in patients with B cell malignancies [57]. This trial suggests ARQ531 could be an effective therapeutic option for patients with relapsed or refractory B cell malignancies, including BTK C481S mutated CLL cases that are resistant to covalent BTK inhibitors. The phase I b expansion portion at 65 mg daily of this study is ongoing, and updated data are pending [57].

ARQ 531 may be also effective in other types of hematological malignancies. ARQ 531 could target multiple pathways including BTK, MYB, AKT, ERK, and other pathways in acute myeloid leukemia (AML) [58]. In preclinical models of AML, ARQ 531 showed in vitro and in vivo activities against AML [59]. ARQ 531 was demonstrated to be synergistic with venetoclax in AML xenograft model [59], suggesting the combination of ARQ 531 with venetoclax could be a potential therapeutic option in the treatment of AML.

#### LOXO-305

LOXO-305 is a next-generation, reversible BTK inhibitor, which potently inhibits both WT and C481S mutant BTK with nanomolar potency and shows high selectivity with minimal off-target inhibition [60]. LOXO-305 potently inhibits Y223 autophosphorylation of all active BTK mutants (BTK C481S, C481T, and C481R). In vitro studies showed that LOXO-305 potently led to inhibition of BCR signaling and cell survival in both treatment-naive and BTK C481 mutant CLL primary cells, indicating that LOXO-305 could be used for treating treatment-naïve and ibrutinib-resistant CLL patients [60–62]. In high proliferating tumors, high rates of BTK turnover may result in incomplete target inhibition by covalent inhibitors, which ultimately lead to resistance to these covalent BTK inhibitors. LOXO-305 achieves remarkable target coverage even in the presence of high rates of BTK turnover, providing a rationale for using LOXO-305 in aggressive B cell lymphomas including DLBCL.

A multicenter phase I/II BRUIN trial evaluating oral LOXO-305 in patients with previously treated B cell malignancies is currently ongoing [63, 64]. Preliminary results were reported at the 2020 ASH meeting [63, 64]. A total of 186 patients include 94 patients with CLL/SLL, 38 with MCL, 19 with DLBCL, 17 with WM, 6 with FL, 5 with MZL, and 7 patients with other (B-PLL and Richter’s transformation) [63]. A 3 + 3 dose-escalation design was used, and patients were treated on 7 dose levels (25 mg to 300 mg QD) [15]. The enrolled patients were heavily treated, and the median number of prior therapies was 4 for CLL/SLL (range 1–10), 2 for MCL (range 2–8), and 3 for other NHLs (range 2–11). In total, 84% of CLL/SLL patients were previously treated with a BTK inhibitor and 31% venetoclax. And 92% of MCL patients had received a prior BTK inhibitor [64]. LOXO-305 demonstrated high oral bioavailability, with doses ≥ 100 mg QD resulting in higher than 90% of the maximum inhibition for the entire dosing interval [63]. There was no dose-limiting toxicity or dose reductions. The only emergent AEs related to LOXO-305 were fatigue (*n* = 29, 16%) and diarrhea (*n* = 28, 15%). A recommended phase 2 dose of 200 mg QD was selected as the recommended dose for future studies. The clinical activity of LOXO-305 was demonstrated within the first cycle of therapy and at the first dose level. Among the 94 CLL/SLL patients, 88 patients remained on therapy. And among the 65 CLL/SLL patients that were efficacy-evaluable (58 BTK inhibitor-treated, 7 BTK inhibitor-naïve), the overall response rate (ORR) was 57% with 23 PRs and 14 PR-Ls [63]. The therapeutic efficacy of LOXO-305 in MCL patients was also remarkable. Among 35 efficacy-evaluable MCL, the ORR was 51% with 9 CRs and 9 PRs [64]. For other NHL patients, 15 DLBCL patients (ORR: 20%, with 3 CRs), 5 FL patients (ORR: 60%, with 3 PRs), 3 MZL patients (ORR: 67%, with 2 PRs), and 6 other patients (ORR: 33%, with 2 PRs) were efficacy-evaluable [64]. This study suggested that LOXO-305 was well-tolerated and was effective in patients with heavily pretreated CLL/SLL and NHLs, including those who had developed resistance to ibrutinib and venetoclax. However, longer follow-up and a larger number of patients are needed to determine its efficacy and safety.

### Other non-covalent BTK inhibitors

In addition to the several non-covalent BTK inhibitors that have shown promising efficacy in clinical trials, there are also some other non-covalent BTK inhibitors with significant antitumor effects in preclinical studies (Table 4). For instance, XMU-MP-3, a non-covalent inhibitor with potent BTK inhibitory activity, inhibited B cell lymphoma cells with or without BTK C481S mutation in vitro and in vivo [65], suggesting it could be effective in treating B cell lymphomas including those resistant to ibrutinib. Several other non-covalent BTK inhibitors including CB1763, GNE-431, and CGI-1746 have shown potent inhibitory effects on both wildtype and C481S mutant BTK [66, 67]. Further clinical trials are warranted to investigate the safety and efficacy of these novel agents.

**Table 4:** Representative non-covalent BTK inhibitors in preclinical stage

Novel non-covalent BTK Inhibitor	Chemical structure	Molecular Formula	In vitro	In vivo	References
XMU-MP-3		C_27_H_27_F_3_N_8_O	XMU-MP-3 is a potent BTK inhibitor with IC50 of 10.7 nM for WT BTK. It also effectively inhibits C481S mutant BTK in vitro. XMU-MP-3 suppresses the proliferation of BTK-transformed Ba/F3 cell with an IC50 of 11.4 nM. It remains active against C481S mutant BTK-transformed Ba/F3 cells with an IC50 of 182.3 nM	XMU-MP-3 remarkably inhibits tumor growth in BTK-transformed Ba/F3 and Ramos in mouse xenograft models without affecting animal weights	[65]
CB1763 (also known as AS-1763)	NA	NA	CB1763 potently, reversibly inhibits both WT and C481S mutant BTKs (IC50 = 0.85 and 0.99 nM for WT and C481S, respectively). CB1763 substantially reduces BTK Tyr223 autophosphorylation at nanomolar concentration in HEK293 cells that are transfected with C481S mutant BTK	CB1763 shows excellent antitumor activity in the BTK-driven OCI-Ly10 xenograft model	[92]
GNE-431		C_30_H_32_N_10_O_2_	GNE-431 potently inhibits WT BTK and C481S mutant BTK (IC50 = 3.2 and 2.5 nM for WT and C481S mutant, respectively). Additionally, GNE-431 shows high potency against several other BTK mutants including C481R, T474I, and T474M mutants (IC50 = 7.5–10 nM). GNE-431 potently suppresses BTK autophosphorylation in C481S BTK mutant-transfected cells	No in vivo data regarding the activity of GNE-431 in B cell malignancies to date	[66]
CGI-1746		C_34_H_37_N_5_O_4_	By occupying an H3 binding pocket, CGI-1746 stabilizes an inactive conformation of BTK. CGI-1746 reversibly and selectively inhibits BTK with IC50 of 8.9 nM and 16 nM for WT BTK and C481S mutant, respectively. CGI-1746 induces significant cell cycle arrest of Ramos cells in vitro	No in vivo data regarding the activity of CGI-1746 in B cell malignancies to date	[67, 93]

*BTK* Bruton tyrosine kinase, *IC50* half-maximal inhibitory concentration, *nM* nmol/l, *WT* wildtype, *NA* not available

## Future directions

Although some non-covalent BTK inhibitors have shown promising efficacy in the treatment of relapsed or refractory B cell malignancies, the currently available data are all from phase I or II studies with a small number of participants. Phase II studies that have larger enrollment are needed to verify the safety and efficacy of non-covalent BTK inhibitors. In addition to patients who have developed resistance to covalent BTK inhibitors, patients who are unable to tolerate covalent BTK inhibitors may also benefit from non-covalent BTK inhibitors, which remains to be verified. It is also important to conduct phase III trials to compare non-covalent BTK inhibitors with covalent BTK inhibitors in the treatment of B cell malignancies. As the combinations of ibrutinib with immunochemotherapy or venetoclax have resulted in high rates of MRD negativity in patients with untreated CLL [20, 21], it would be interesting to see whether combining non-covalent BTK inhibitors with immunochemotherapy or venetoclax produces similar effects in patients with CLL.

In addition to non-covalent BTK inhibitors, proteolysis targeting chimeras (PROTACs) are also designed to target BTK and BTK mutants [68]. PROTACs simultaneously bind to an E3 ligase and a target protein, thereby resulting in ubiquitination and subsequent degradation of the target protein. MT-802 is a PROTAC that recruits BTK to the cereblon E3 ligase complex and triggers ubiquitination and subsequent degradation of both wild-type and C481S mutant BTK, thereby providing a rationale for using PROTACs to treat B cell malignancies including those resistant to ibrutinib due to BTK C481S mutation [69]. Another PROTAC P13I could remarkably inhibit the proliferation of BTK C481S mutant DLBCL cell line HBL-1, which is resistant to ibrutinib [70], providing a potential treatment for ibrutinib-resistant B cell malignancies. Several other PROTACs have also shown potential therapeutic efficacy for BTK C481S mutant B cell malignancies in preclinical models [71]. Clinical trials are required to explore the safety and efficacy of PROTACs in patients with B cell malignancies including those resistant to ibrutinib.

## Conclusions

BTK, the essential component of BCR signaling, plays a vital role in the origin and development of B cell malignancies. The covalent BTK inhibitors, especially ibrutinib, acalabrutinib, and zanubrutinib, bring benefits for patients with CLL and other B cell malignancies. With resistance and off-target toxicities caused by covalent inhibitors, preclinical and clinical studies of non-covalent BTK inhibitors are ongoing. To date, several non-covalent BTK inhibitors studied in B cell malignancies have shown great specificity and mild AEs in clinical trials, while long-term efficacy and safety remain uncertain; and the others in preclinical studies need further evaluation before clinical trials. Compared to covalent inhibitors, non-covalent inhibitors display prominent advantages when treating patients with BTK C481 mutations. Nonetheless, further clinical and preclinical studies of non-covalent BTK inhibitors are essential to evaluate the specificity, efficacy, and safety.

## Data Availability

Not applicable.

## Abbreviations

BCR: B cell receptor
BTK: Bruton tyrosine kinase
SYK: Spleen tyrosine kinase
PI3K: Phosphatidylinositol-3-kinase
ITAM: Immunoreceptor tyrosine-based activation motif
NF-κB: Nuclear factor κB
ITK: Interleukin-2-inducible T cell kinase
TEC: Tyrosine kinase expressed in hepatocellular carcinoma
RLK: Resting lymphocyte kinase
BMX: Bone marrow expressed kinase
CLL: Chronic lymphocytic leukemia
SLL: Small lymphocytic lymphoma
MCL: Mantle cell lymphoma
WM: Waldenström's macroglobulinemia
MZL: Marginal zone lymphoma
FL: Follicular lymphoma
DLBCL: Diffuse large B cell lymphoma
RT: Richter transformation
AE: Adverse event
MRD: Minimal residual disease
PLCG2: Phospholipase C-gamma-2
MTD: Maximum tolerated dose
ASH: American Society of Hematology
CR: Complete response
PR: Partial response
PR-L: PR with lymphocytosis
WT: Wild type
ORR: Overall response rate
PROTACs: Proteolysis targeting chimeras
